# pH-Sensitive Liposomes for Enhanced Cellular Uptake and Cytotoxicity of Daunorubicin in Melanoma (B16-BL6) Cell Lines

**DOI:** 10.3390/pharmaceutics14061128

**Published:** 2022-05-26

**Authors:** Hamad Alrbyawi, Ishwor Poudel, Manjusha Annaji, Sai H. S. Boddu, Robert D. Arnold, Amit K. Tiwari, R. Jayachandra Babu

**Affiliations:** 1 Department of Drug Discovery and Development, Harrison School of Pharmacy, Auburn University, Auburn, AL 36849, USA; hrbyawi@taibahu.edu.sa (H.A.); izp0018@auburn.edu (I.P.); mza0153@auburn.edu (M.A.); rda0007@auburn.edu (R.D.A.); 2 Pharmaceutics and Pharmaceutical Technology Department, College of Pharmacy, Taibah University, Medina 41477, Saudi Arabia; 3 Department of Pharmaceutical Sciences, College of Pharmacy and Health Sciences, Ajman University, Ajman P.O. Box 346, United Arab Emirates; 4 Center of Medical and Bio-Allied Health Sciences Research, Ajman University, Ajman P.O. Box 346, United Arab Emirates; amit.tiwari@utoledo.edu; 5 Department of Pharmacology and Experimental Therapeutics, The University of Toledo, Toledo, OH 43614, USA

**Keywords:** pH-sensitive, liposomes, melanoma, cardiolipin, daunorubicin

## Abstract

Daunorubicin (DNR) was delivered using a pH-sensitive liposomal system in B16-BL6 melanoma cell lines for enhanced cytotoxic effects. DNR was encapsulated within liposomes and CL as a component of the lipid bilayer. PEGylated pH-sensitive liposomes, containing CL, were prepared in the molar ratio of 40:30:5:17:8 for DOPE/cholesterol/DSPE-mPEG (2000)/CL/SA using the lipid film hydration method and loaded with DNR (drug: lipid ratio of 1:5). The CL liposomes exhibited high drug encapsulation efficiency (>90%), a small size (~94 nm), narrow size distribution (polydispersity index ~0.16), and a rapid release profile at acidic pH (within 1 h). Furthermore, the CL liposomes exhibited 12.5- and 2.5-fold higher cytotoxicity compared to DNR or liposomes similar to DaunoXome^®^. This study provides a basis for developing DNR pH-sensitive liposomes for melanoma treatment.

## 1. Introduction

The American Cancer Society predicted that 99,780 new cases of melanoma would occur in 2022 (7% of all cancer cases). Of those cases, 7650 patients are expected to die, predominantly because of widespread metastases Patients with stage IV disease, where cancer has metastases in distant visceral sites, have a 1-year survival rate of 41% [1]. Although treatment of primary cutaneous melanoma by surgery yields a high survival rate, advanced metastatic melanoma cannot be treated by surgery alone and, thus, requires better therapeutic methods [2]. Since radiation therapy alone has also proven to be ineffective for treating metastatic melanoma, chemotherapeutic drugs such as dacarbazine and temozolomide are being used to medically manage metastatic melanomas [3].

Chemotherapeutic agents have been used for the treatment of metastatic melanoma for over three decades. However, the modest antitumor activity of the cytotoxic drugs led to the investigation of combinations of these agents to improve therapeutic outcomes [4]. Combinations of cytotoxic drugs may yield higher response rates than monotherapy but are associated with more significant side effects such as hematologic, gastrointestinal, and cutaneous toxicities [5]. Targeted drug delivery devices are typically more effective than conventional treatments and usually exhibit fewer side effects and less systemic toxicity [6]. These devices usually carry multiple components, such as targeting agents, imaging agents, and anticancer drugs, for optimized functions such as drug targeting to the tumor site and easy diagnosis.

The use of liposomes as drug delivery systems was highly successful due to their ability to enhance the solubility of poorly soluble drugs and encapsulate a wide range of drugs. Besides, their efficiency, biocompatibility, and nonimmunogenicity have increased their use as drug delivery systems [7]. Liposomes consist of a lipid bilayer with an aqueous phase where hydrophilic moieties can be entrapped while hydrophobic moieties can be localized into the bilayer membrane [8]. Liposomes’ properties differ considerably across lipid compositions since the lipid bilayer components determine their rigidity, size, release rate, and surface charge. For instance, saturated phospholipids with long acyl chains (for example, 1,2-dipalmitoyl-sn-glycero-3-phosphocholine, DPPC) form a rigid and more stable bilayer structure compared to unsaturated phosphatidylcholine types. The incorporation of the 1,2-distearoyl-sn-glycero-3-phosphoethanolamine-N-amino-polyethyleneglycol-2000 (DSPE-mPEG-2000) lipid into the liposomes’ bilayer is critical for the prolongation of liposome circulation time in the bloodstream [9]. Another rationale for the use of PEGylated liposomes for DNR is to reduce cardiotoxicity and decrease gastrointestinal side effects such as nausea and vomiting. PEGylated liposomes decreased cardiotoxicity due to the targeted delivery of anthracycline drugs and reduced the tendency to accumulate in the cardiac tissue [10]. Major obstacles to liposomal drug delivery are slow drug release and the absence of fusogenic activity after internalization into the endosomal compartment [11].

The development of pH-sensitive liposomes is a very promising strategy for cancer treatment. The concept is based on the fact that tumors usually have a lower pH than healthy tissue, and stimuli-sensitive liposomes can be prepared to release the incorporated drug only when subjected to this unique tumor condition [12]. pH-sensitive liposomes have been designed to be stable at physiological pH, but to be destabilized upon acidification by the tumor microenvironment, thereby promoting the release of their encapsulated contents [13]. Acidic extracellular pH is a major characteristic of tumor tissue, largely considered to be due to lactic acid secretion from anaerobic glycolysis in hypoxia and an excess amount of CO2 production [14,15]. In the tumor microenvironment, a local pH range from 5.5 to 7.0 is not unusual [16].

1,2-dioleoyl-sn-glycero-3-phosphoethanolamine (DOPE) is one of the critical components of pH-sensitive liposomes. When liposomes containing DOPE are incubated in acidic pH, they undergo destabilization. This effect is facilitated by low hydration of the polar head group of DOPE, which is converted to a hexagonal inverted phase causing the formation of non-lamellar structures that trigger destabilization of liposomes bilayers at acidic pH [17]. pH-sensitive lipid DOPE has a strong propensity to form a nonbilayer structure at acidic pH, causing liposomes to release their contents in response to acidic pH in the tumor microenvironment while remaining stable in plasma, thus enhancing the cytoplasmic delivery of different agents [18].

Non-bilayer lipids such as DOPE, which have a cone shape and will not form the bilayer alone but can be stabilized in a bilayer structure by the incorporation of a bilayer, prefer lipids such as phosphatidylserine (PS) or a weakly acidic amphiphile such as cholesteryl hemisuccinate (CHEMS) [19]. The incorporation of stabilizing lipids causes liposomal formulations to be stable at neutral pH. Under acidic conditions, the stabilizing lipid becomes partially protonated and loses its ability to stabilize the bilayer structure. Reducing the stabilizing effect as a result of protonation of the stabilizing lipid will allow DOPE molecules to revert into their inverted hexagonal phase [20], thus the destruction of the liposomal bilayer organization and payload release.

Among phospholipid classes, Cardiolipin (CL) has an interesting chemical structure, being highly acid and having a head group (glycerol) that is esterified to two phosphatidylglyceride backbone fragments instead of one, forming a dimeric structure [21]. CL lipid is crucial for both mitochondrial bioenergetics and many cellular processes outside of the mitochondria, such as cell apoptosis and cell wall biogenesis [22]. Due to its unique structure, CL can have a nonbilayer propensity in the context of biomembranes, promoting local regions of high curvature because it forms inverted hexagonal structures in isolation under certain conditions such as low pH [23]. CL increases the bilayer fluidity as its presence introduces a higher unsaturation degree to the membrane bilayer [24]. Furthermore, when CL interacts with calcium across the membrane, it leads to changes in lipid packing and structure, increasing the flip-flop motion of lipids [25]. CL has some effects on the mechanical properties of the membrane. CL decreases the mechanical stability of the membrane due to a decrease in lipid packing and the formation of nonlamellar structures, resulting in the deformation of the biological membrane [26].

Daunorubicin (DNR), an anthracycline derivative, is a potent chemotherapy drug that exhibits broad-spectrum anti-tumor activity against a wide range of cancers, including blood malignant cancers (such as leukemia and lymphoma) and many types of solid (carcinoma) and soft (sarcomas) tissue tumors [27]. It produces its anti-tumor activity by blocking topoisomerase 2, an enzyme that cancer cells need in order to divide and grow [28]. Due to poor targeting efficiency, DNR has many side effects such as cardiotoxicity, acute vomiting and nausea, gastrointestinal problems, baldness, and disturbances to the neurological system [29]. Compared with conventional anthracyclines, liposomal formulations of anthracyclines exhibit less toxicity because injected liposomes cannot pass the vascular space in sites that have tight capillary junctions, such as the heart muscle [30]. Due to their ability to deliver drugs to their intended site of action, liposomal formulations’ antitumor efficacy is better or at least comparable to that of the conventional formulations [8].

The objective of this study was to determine the cytotoxicity and cellular uptake of pH-sensitive DNR liposomal formulation enriched with CL. Many model membrane studies determined that the incorporation of CL leads to conformational changes in the membrane structure, making the membrane structurally deformed and more permeable [24].

## 2. Materials and Methods

### 2.1. Materials

1,2-dioleoyl-sn-glycero-3-phosphoethanolamine (DOPE), 1,2-distearoyl-sn-glycero-3-phosphoethanolamine-N-[methoxy(polyethylene glycol)-2000] (ammonium salt) (DSPE-mPEG2000), cardiolipin (CL), 1,1’,2,2’-tetraoleoyl cardi-olipin [4-(dipyrrometheneboron difluoride)butanoyl] (ammonium salt), TopFluor^®^ CL, and cholesteryl hemisuccinate (CHEMS) were purchased from Avanti Polar Lipids Inc (Ala-baster, AL, USA). Stearylamine (SA) was purchased from Sigma- Aldrich (St. Louis, MO, USA). Cholesterol and ammonium sulfate were purchased from JT Baker (Phillipsburg, NJ, USA). Fetal bovine serum (FBS), Dulbecco’s Modified Eagle’s Medium (DMEM), Earle’s Balanced Salt Solution (EBSS), and other reagents for cell culture were purchased from Mediatech (Manassas, VA, USA). Daunorubicin was purchased from AvaChem Scientific (San Antonio, TX, USA). 2′,7′-Dichlorofluorescin diacetate and Phosphate-Buffered Saline pH 7.4 (PBS) were purchased from Sigma-Aldrich (St. Louis, MO, USA). The bicinchoninic acid protein kit was purchased from Thermo Fisher Scientific (Hanover Park, IL, USA). 3-(4,5-Dimethyl-2-thiazolyl)-2,5-diphenyl-2H-tetrazolium bromide (MTT) was purchased from Calbiochem (Darmstadt, Germany). Polycarbonate membrane (0.08 μm) was purchased from Whatman Maidstone, UK). Melanoma (B16BL6) cancer cells were obtained from the American Type Culture Collection (Manassas, VA, USA).

### 2.2. Liposomes Preparation

Liposomes were prepared by the lipid film hydration technique using a rotary vacuum evaporator. Briefly, DOPE, cholesterol, DSPE-mPEG2000, CL, and SA were prepared as 10 mg/mL solutions individually in chloroform. These solutions were mixed at a molar ratio of 40:30:5:17:8 for DOPE/cholesterol/DSPE-mPEG (2000)/CL and SA. The mixture was flash evaporated on a Rotavapor (Büchi, Germany) by applying approximately 25 mmHg vacuum at 65 °C water bath temperature. The lipid film deposited on the wall of the flask was further dried under a stream of nitrogen for 1 h, followed by vacuum desiccation for 2 h. The dry lipid film was then hydrated in a 250 mM ammonium sulfate solution (pH 5.5). This mixture was then placed in a water-bath incubator (65 °C) for 1 h to form coarse liposomes. This mixture was then subjected to seven liquid nitrogen freeze–thaw cycles above the phase transition temperature of the different lipids before extrusion. The liposome mixture was then extruded through an 80 nm (10 passes) polycarbonate filter using the Lipex^®^ 10 mL barrel extruder (Transferra Nanosciences Inc., Burnaby, BC, Canada). The free ammonium sulfate outside the liposomes was removed by dialysis (using 12,000 to 14,000 Daltons molecular weight cut off dialysis tubing) against a sucrose solution (10% *w*/*v*, 250 mL) at 4 °C. The solution medium was then discarded and replaced with a fresh solution at 1, 4, and 8 h intervals and then left overnight. The phospholipid concentration of each formulation was quantified following acid hydrolysis and an inorganic phosphate assay [31]. A liposomal formulation similar to DaunoXome^®^, composed of DSPC/cholesterol/daunorubicin (in a 10:5:1 molar ratio), was prepared by the same method; however, citrate was used instead of ammonium sulfate to hydrate the lipid film. Table 1 summarizes the different formulations prepared.

### 2.3. Drug Encapsulation in Liposomes (Active Loading)

A DNR solution of an appropriate concentration was prepared by adding the required quantities of the drug in the PBS, and this drug solution, after adjusting the pH to 8 with 0.1 N NaOH, was added to the lipid solution at appropriate drug-to-lipid ratios (1:5). Excess DNR was then removed by dialysis against the sucrose solution (10%) at 4 °C. Based on the initial results of the drug loading efficiency, a 1:5 drug-to-lipid ratio was found to be optimum, and this ratio was used for all formulations. DNR was added as a solution, after adjusting its pH to 8 with 0.1 N NaOH, to the liposome dispersion to give a drug-to-lipid ratio of 1:5. The loading was performed at 65 °C for 1 h. Excess DNR was then removed by dialysis against sucrose solution (10%) at 4 °C using dialysis tubing (regenerated cellulose, molecular weight cut off 12,000 Da, Fisher Scientific, Suwanee, GA, USA).

### 2.4. Encapsulation Efficiency (EE%) and Drug Loading (DL%) Measurement

The amount of DNR entrapped in the liposomes (EE% and DL%) was determined fluorometrically at 480 nm (excitation) and 590 nm (emission) using a microplate reader 142 (Fluostar, BMG Labtechnologies, Ortenberg, Germany). Briefly, Triton X-100 (1%) was added to the liposomal DNR to break the liposome bilayer and release the entrapped DNR. The liposomal drug concentration was calculated from a DNR standard curve. All experiments were run in triplicate and mean data were presented.

The EE% was calculated as follows:

$$Encapsulation\;Efficiency\;\left(\%\right)=\frac{\left(amount\;of\;liposomal\;drug\right)}{total\;amount\;of\;drug}\times 10\;$$

The DL % was calculated as follows:

$$Drug\;Loading\;\left(\%\right)=\frac{\left(amount\;of\;liposomal\;drug\right)}{total\;amount\;of\;drug\;added+amount\;of\;excipients\;added}\times 100\;$$

### 2.5. Determination of Particle Size and Zeta Potential of Liposomal Formulations

The particle size distribution of the liposomal formulations was carried out by the dynamic light scattering method using a Nicomp 380 ZLS particle size analyzer (Particle Sizing Systems, Santa Barbara, CA, USA). The mean particle size and polydispersity index of the formulations after appropriate dilutions were calculated. The zeta potential of liposome measurements was determined by photon correlation spectroscopy (PCS, Zetatrac, Largo, FL, USA). For the analyses, the formulations were diluted in an aqueous medium. All determinations were performed in triplicate at room temperature (25 °C).

### 2.6. In Vitro Release Studies

The release of DNR from liposome formulations was determined by the dialysis method. PBS (pH = 7.4) and PBS (pH = 5.5) filled in 250 mL conical flasks, were used as a receptor phase. Regenerated cellulose dialysis tubing (with 12,000 to 14,000 Daltons for the molecular weight cut off), with a 30 mm × 25 mm release area, pre-soaked in buffer solution for one hour, was used. One milliliter of the formulation of the DNR solution was placed in the dialysis tubing, which was immersed in the receptor phase. All flasks were incubated at 37 °C in a rotary shaker set at 150 rpm. Samples (1 mL) were collected at different time intervals, and the sample volumes were replenished with fresh buffer immediately. The concentration of DNR in the receptor buffer (dialysate) was analyzed fluorometrically at 480 nm (excitation) and 590 nm (emission) using a microplate reader 142 (Fluostar, BMG Labtechnologies, Ortenberg, Germany). The cumulative amount of DNR released versus time was plotted. Experiments were run in triplicate and mean data were presented.

### 2.7. Stability Studies

Stability studies were conducted to monitor the physical stability of the liposomes. All liposomal formulations were stored at 4 °C under N_2_ and protected from light for up to one month, and EE, particle size, zeta potential, and polydispersity were determined after performing dialysis to remove the non-capsulated drug.

### 2.8. Cell Culture

Melanoma (B16-BL6) cells were cultured in Dulbecco’s Modified Eagle’s Medium (DMEM). The medium was supplemented with 10% fetal bovine serum (FBS), 100 U/mL penicillin, and 100 μg/mL streptomycin at 37 °C in a humidified atmosphere containing 5% CO_2_. All experiments were performed at a confluence of 90 to 95%. The pH of the cell culture medium was measured to determine the pH of the extracellular fluid.

### 2.9. Measurement of Cell Viability by MTT Assay

B16-BL6 cells were cultured in flat-bottom 96-well plates for 24 h. The cell density in the wells was approximately 8 × 10^3^ cells/well. The cells received treatments of various liposomal formulations (0.01 μM, 0.05 μM, 0.1 μM, 0.5 μM, 1 μM, and 2 μM) for 48 h prior to the MTT assay. After treatments, 10 μL of 3-[4, 5-dimethylthiazol-2-yl]-2, 5-diphenyl tetrazolium bromide (MTT) was added to each well and the cells were incubated at 37 °C for an additional 2 h. Finally, the medium was aspirated, and 200 μL dimethylsulfoxide (DMSO) was added to each well to solubilize the dye remaining in the plates. The absorbance was measured using a microplate reader (Spectramax M5, molecular devices, Sunnyvale, CA, USA) at 544 nm. All the experiments were run in triplicate and mean data were presented.

### 2.10. Cellular Daunorubicin Uptake

B16-BL6 cells were cultured in flat-bottom 24-well plates. At the optimum confluence, the cells were exposed to 14 μM liposomal DNR or free DNR for 4, 8, and 12 h. After extensive washing with PBS, cells were lysed in 100 μL of 1% Triton X-100. DNR fluorescence was then measured by a microplate reader (Spectramax M5, molecular devices, Sunnyvale, CA, USA) at 480 and 590 nm for excitation and emission, respectively. Cellular DNR contents were calculated and corrected for any differences in protein content, as determined with the bicinchoninic acid assay [32]. All values were corrected for background fluorescence. All experiments were run in triplicate, and mean data were presented.

### 2.11. Daunorubicin Retention Studies

To evaluate the cellular DNR accumulation by cancer cells after the drug efflux period, cells grown in 24-well plates were loaded with DNR in the form of free DNR solution or liposomal-DNR (14 μM) for four h. The supernatant was removed at the end of treatment, and cells were washed with ice-cold PBS. The wells were refilled with fresh drug-free EBSS, and cells were incubated at 37 °C to facilitate cellular drug efflux. At predetermined time intervals (1, 2, and 4 h), the supernatant containing the effluxed drug was removed. Cells were washed and lysed, and the amount of DNR retained by the cells was measured with a microplate fluorometer as described above.

### 2.12. Fluorescence Microscopy

B16-BL6 cells were seeded in a flat-bottom 24-well plate for 24 h. After exposure to liposomal DNR or free DNR for 14 h, cells were washed and fixed [15 min in 4% (*w*/*v*) paraformaldehyde in phosphate-buffered saline]. All samples were examined with a fluorescence microscope (EVOS fl, ZP-PKGA-0494 REV A, Waltham, MA, USA) and photographed at 20× magnification.

### 2.13. Statistical Analysis

All the data were presented as mean ± standard deviation. GraphPad Prism software was used to determine the statistical levels of significance. All data were subjected to a one-way analysis of variance (ANOVA) followed by Tukey’s multiple comparison test. A *p*-value less than 0.05 was considered to be statistically significant.

## 3. Results and Discussion

The liposomes were evaluated for EE% and DL%. To obtain liposomes with desirable EE%, DNR was mixed with the lipid (DNR:lipid) at a ratio of 1:5. DNR was loaded into the aqueous phase of the liposome by active loading, using ammonium sulfate 250 mM. As shown in Table 2, the EE% and DL% of the formulations were above 90 and 15, respectively. Our preliminary studies showed that a 1:5 drug-to-lipid ratio demonstrated a higher EE% and DL%, hence this ratio was used for all liposomal formulations. The drug-to-lipid ratio has a significant influence on the EE of DNR. An indirect relationship has been observed between EE% and the drug concentration. The EE% decreases with an increased drug concentration [33]. The existence of drug precipitate in the liposome interior may explain the inverse relationship between EE and drug concentration. Increasing the drug-to-lipid ratio beyond 1:5 causes the drug to precipitate inside the liposomes, leading to significant disruption of the liposomal membrane, which causes leakage of the encapsulated drug from liposomes [34].

High EE% of amphipathic weak bases, such as DNR, might be achieved by a transmembrane ammonium sulfate gradient in and out of liposomes (active loading) [35,36]. As with many drugs, DNR was not efficiently entrapped in the aqueous phase of the liposome without a pH gradient [37]. In the active loading method, liposomes are initially prepared in an acidic environment. After vesicle self-assembly, the core of the liposome remains acidic while the extravesicular pH level is similar to physiological conditions [38]. Remote loading of the uncharged drug allows molecules to diffuse into the liposomal intravesicular interior where they become protonated. The positively charged drug can no longer cross the bilayer membrane and is trapped inside the liposome structure [39].

To increase the percentage of the drug encapsulated inside liposomes, we replaced cholesterol with CHEMS. The DOPE lipid by itself, with these structural aspects (inverted hexagonal phase), cannot form lipid bilayers at neutral pH, so it must be combined with amphiphilic molecules containing a protonatable acidic group such as CHEMS to form bilayers [40]. The insertion of PEG on the surface of liposomes is a common strategy to enhance the hydrophilicity of the particle surface, as the reticuloendothelial system (RES) preferentially takes up particles with a hydrophobic surface [41]. It is important to mention that mole% PEG can significantly affect the percentage of the drug encapsulated inside liposomes. An inverse relationship was noticed between the mole% of PEG and EE of drugs since PEG might occupy space in the core of the liposomes [42]. The mole % of PEG used in our formulation does not affect DNR EE. There is no statistically significant difference in EE between F1 and F2 (*p* > 0.05).

The particle size of F1 was approximately 94 nm with a polydispersity of 0.2, indicating uniform and dispersed liposomal formulations (Table 2). The zeta potential of F1 was negative because CL is a quadruple-chained anionic amphiphile lipid composed of two 1,2-diacyl phosphatidate moieties esterified to the 1- and 3-hydroxyl groups of a single glycerol molecule. Under physiological conditions, phosphodiester moieties should both be negatively charged [43]. Besides, the PEG-DSPE lipid, incorporated into liposomes to extend the circulation time, imparts a negative charge [44]. The zeta-potential of F2 was in the neutral range since it is composed of neutral lipids.

As shown in Table 2, there is no significant difference in particle size between liposome formulations with or without CL, indicating that the addition of CL does not affect particle size (*p* > 0.05). Particle size is a significant parameter that plays a crucial role in the pharmacokinetics of drug distribution. The liver, spleen, and other parts of the RES usually take up liposomes larger than 200 nm [45]. Therefore, liposomes less than 200 nm in diameter and of uniform size are preferred for tumor targeting [46]. The liposome preparation method allowed the instantaneous and reproducible formation of DNR-CL liposomes with a mean particle size below 100 nm in size and high entrapment efficiency (>90%).

**Figure 1:**
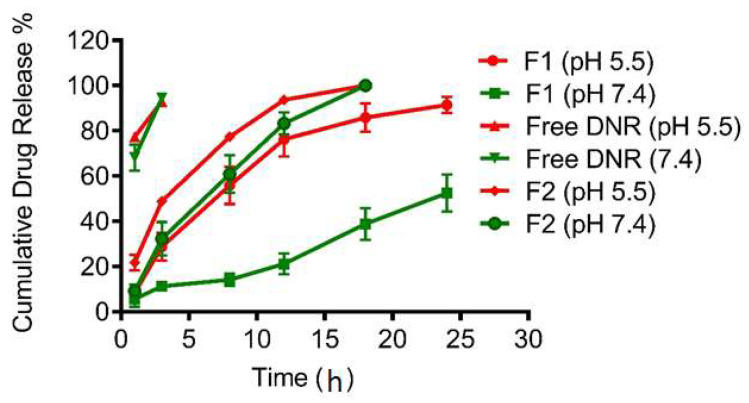
In vitro release profiles of DNR encapsulated liposomes. Values represent Mean ± SD, *n* = 3.

Figure 1 shows the DNR release from the dialysis studies on free DNR, F1, and F2 at pH 5.5 as well as pH 7.4. Free DNR as a solution diffused rapidly and was completely (100%) released after 1–2 h. At pH 7.4, F1 liposomes showed only 50% DNR release within 24 h while F2 liposomes released DNR rapidly (50% of DNR was released within 5 h). There was no significant change in the release profile for both free DNR and F2 liposomes at pH 5.5 as compared to pH 7.4. However, DNR release from pH-sensitive liposomes (F1) was significantly faster at pH 5.5 (50% of DNR was released within 8 h). At pH 7.4, the F1 formulation had a longer release time due to the steric barrier provided by the surface-grafted PEG [47].

Liposomes that exhibit triggered release features have potentially important applications in drug delivery. Liposomes can be formulated to make them sensitive to a variety of physical and chemical conditions, such as temperature, light, or pH [48]. pH-sensitive liposomes have been designed to trigger and promote the fast and efficient release of entrapped molecules in response to an acidic environment. The acidosis in the extracellular microenvironment of tumor tissue can be attributed to the poor organization and dysfunctional vasculature, heterogeneous blood flow, and insufficient nutrient delivery [49].

A DOPE-based, pH-sensitive, liposome drug-delivery system has been widely developed for targeted cancer therapy. Under physiological conditions (pH 7.4), pH-sensitive liposomes exhibit excellent stability; however, in acidic pH (5.5–6.5) DOPE undergoes a phase transition from a lamellar phase to an inverted hexagonal phase (HII), leading to the loss of the spherical structure of the liposomes, and consequently, the release of encapsulated molecules [50]. Lipid components and molar ratios of liposomes (40 mol% DOPE and 5 mol% PEG) in our study achieved the desired release profile consistent with literature data. The leakage of DOX from different pH-sensitive formulations, containing DOPE and CHEMS, was examined with different mol% of DOPE at different pH [51]. Generally, pH-sensitivity improved with increasing mol% DOPE. After incubation for one hour at pH 5.5, the liposomal formulation containing 40 mol% DOPE exhibited pH-sensitivity and enhanced drug release but exhibited good drug retention at pH 7.4. The inclusion of PEG at a mol% similar to our formulation (5%) is not expected to significantly decrease drug release in response to the low pH [52]. As a result, effective release of contents can be achieved by pH-sensitive liposomes while preventing rapid clearance by the RES system.

When we developed the pH-sensitive liposomal DNR formulation, we incorporated CHEMS instead of cholesterol into liposomes in order to obtain the most optimal release profile. CHEMS is an acidic cholesterol ester that self-assembles into bilayers in alkaline and neutral aqueous media. It is commonly used with DOPE to prepare pH-sensitive liposomes as it stabilizes DOPE at a neutral pH and produces intense bilayer deformation when the pH decreases [53]. At a neutral pH, the electrostatic repulsion between deprotonated carboxylate groups of CHEMS and phosphate groups of DOPE allows the formation of bilayer structures. However, at acidic pH, the destabilization of liposomes is facilitated by the protonation of carboxylate groups, eliminating charge repulsion in the bilayer, and subsequently resulting in the reversion of DOPE molecules into their original inverted hexagonal phase [54].

**Figure 2:**
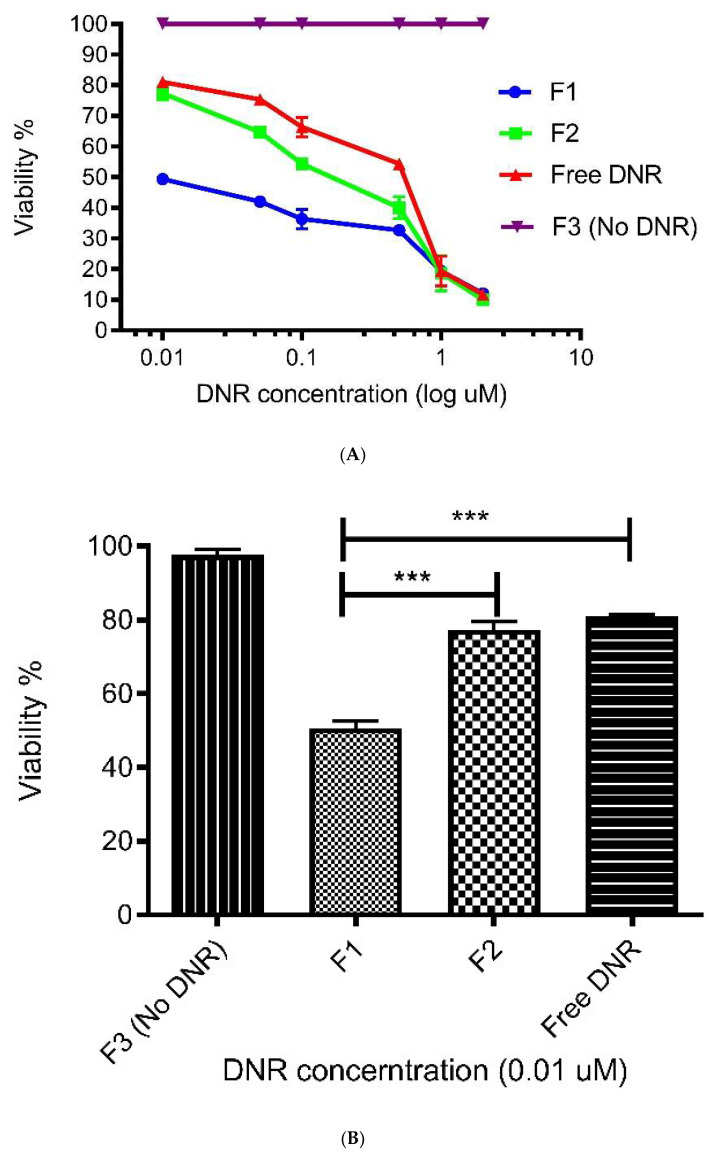
(**A**) CL potentiates the cytotoxic effect of DNR pH-sensitive liposomes against B16-BL6 cell lines. All data are expressed as mean percentages (*n* = 3) compared to untreated control cells. (**B**) In vitro cytotoxicity of different formulations in B16-BL6 cell lines. *** indicates *p* < 0.001. Values represent mean ± SD, *n* = 3.

As shown in Figure 2A,B, the cell viability decreases with an increase in the concentration of DNR in the liposome formulations or DNR solution. The IC_50_ of F1 and F2 were 0.025 and 0.125 µm, respectively, which are approximately 12.5-fold and 2.5-fold lower than the IC50 of the DNR solution (0.3125 µm). The liposome formulation with no DNR (F3) did not show any cytotoxicity, suggesting the formulation and its ingredients are safe for use in liposomes. The lack of specificity of conventional anti-cancer drug dosage forms increases the dose required to reach the target organs to inhibit tumor growth, which explains the high dose of DNR solution required to achieve 50% inhibition [55]. Liposomal drug formulations have been employed to improve the therapeutic efficacy and significantly decrease the toxic effect of anticancer agents, including anthracycline DNR. Furthermore, they impact the pharmacokinetics and tissue distribution of the incorporated anticancer agent [56].

Liposomal anthracyclines were developed to enhance the tumor targeting of conventional anthracyclines to reduce their side effects. Liposomal anthracycline formulations have demonstrated similar efficacy to conventional therapy while improving the safety profile [57]. Various chemical modifications of liposomal formulations have been made (e.g., active targeting) to improve their uptake rate, and consequently, their antitumor activity. The concept of pH-sensitive liposomes emerged from the reality that tumors exhibit an acidic environment as compared with healthy tissues. These liposomes are stable at physiological pH (pH 7.4) but undergo destabilization under acidic conditions, thus leading to the release of their contents.

Many studies have reported that nanoparticle formulations of anthracycline yield less effective in vitro cytotoxicity (i.e., higher IC_50_) than free drugs because nano-particles must release their entrapped drug [58,59]. In our study, the pH of the extracellular fluid of B16-BL6 cell lines ranged between 6.2 and 6.4. In vitro cytotoxic activity of F1 was significantly higher than that of the free solution and F2 (*p* < 0.001). There was no significant difference in cytotoxicity between free DNR solutions and F2 (Figure 2B).

In addition to increasing the release rate of the encapsulated DNR, we altered the permeability of the tumor cell membrane (by using CL) to enhance the therapeutic effect. Remodeling of the cell membrane structure, which comprises both proteins and lipids, is controlled by interactions between specific proteins and lipids. A unique property of the CL lipid is its ability to disturb the packing of the membrane and decrease its mechanical stability. In fact, 5% CL can promote the formation of flowerlike domains that grow with time, leading to membrane structure remodeling, deformation, and permeabilization [24]. In our study, we believe that such physical changes induced by CL might enhance the membrane permeability of DNR, and therefore, its cytotoxic effect. Liposomal formulation enriched with CL resulted in a strong cytotoxic activity in vitro because enhancing membrane permeability will increase the cellular uptake of chemotherapeutic drugs by cancer cells. In our study, the liposomal formulation enriched with CL exhibited enhanced antitumor activity compared to the liposomal formulation without CL. Moreover, the empty liposomal formulation containing CL did not show any effect on cell survival. Thus, CL might contribute to enhancing intracellular DNR delivery. Furthermore, rapid destabilization of pH-sensitive liposomes under acidic pH, in addition to increasing DNR release, might facilitate CL interaction with the membrane.

**Figure 3:**
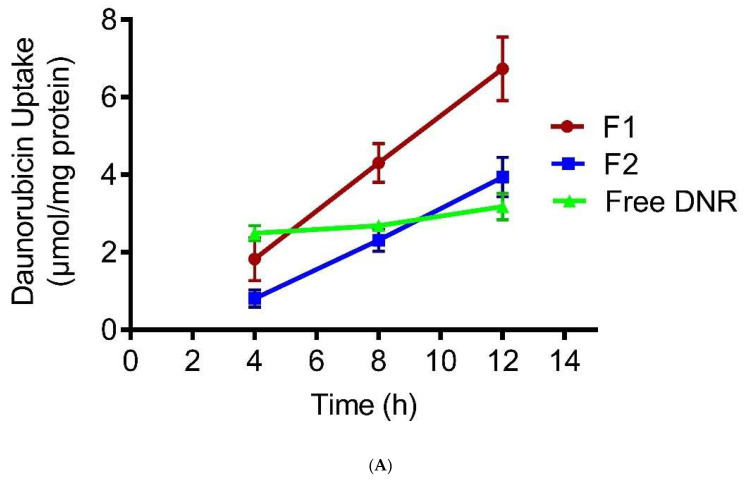

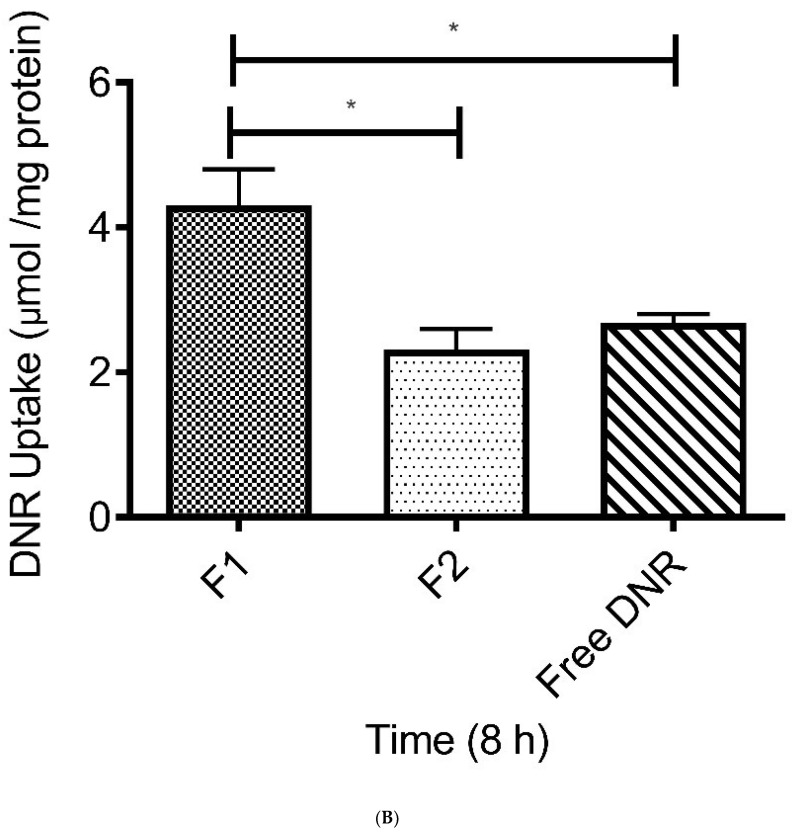
(**A**) Effect of pH-sensitive liposomes enriched with CL on DNR uptake by B16-BL6 cancer cell lines. Values represent mean ± SD, *n* = 3. (**B**). Effect of pH-sensitive liposomes enriched with CL on DNR uptake by B16-BL6 cancer cell lines. * indicates *p* < 0.05. Values represent mean ± SD, *n* = 3.

Figure 3A presents the 12-h time profiles of DNR uptake by tumor cells treated with 14 μM DNR formulations. The cellular DNR levels of the DNR solution reached a plateau within 4 h, whereas F1 cellular DNR levels continued to increase up to 12 h. When treated with DNR solution and F2, B16-BL6 cells accumulated 58% and 44% less DNR at 8 h, respectively, compared to F1 (Figure 3B). There was no significant difference between F2 and free DNR (*p* > 0.05).

Several chemotherapeutic drugs target intracellular organelles, such as the nucleus, to achieve their anticancer activities. For instance, DNR may intercalate between the DNA bases and disrupt the action of topoisomerase II [60]. As a result, effective chemotherapy requires a reasonably high level of drug molecules to accumulate within the cancer cells. Anthracycline drugs enter cells by passive diffusion [61], so their anti-tumor effect can be enhanced either by increasing cellular uptake or increasing cellular retention [62]. In our study, pH-sensitive CL liposomes promoted the rapid release of DNR; however, they exhibited more cellular uptake compared to free DNR. This indicates that incorporating CL plays an important role in increasing cellular uptake. Besides, F1 accumulated higher DNR levels than with F2, which strengthens our hypothesis that CL has an essential role in enhancing cellular uptake.

**Figure 4:**
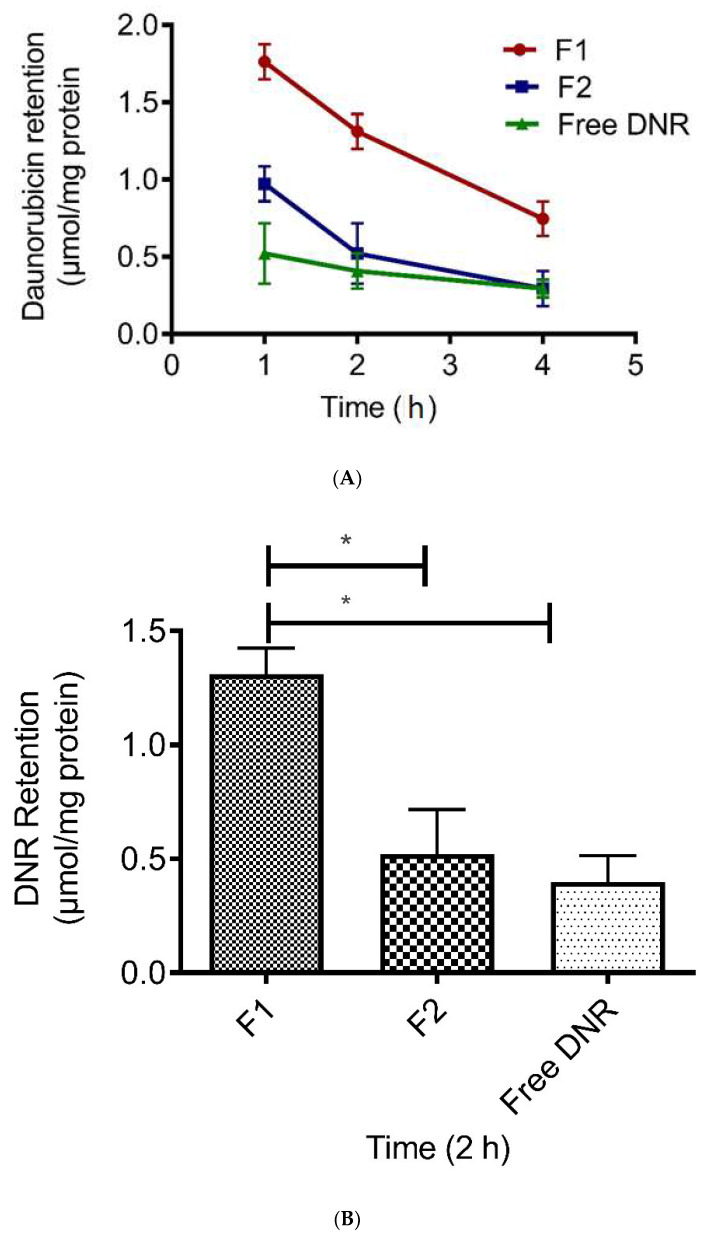
(**A**) Effect of pH-sensitive liposomes enriched with CL on the amount of DNR retained by B16-BL6 cancer cell lines. Values represent mean ± SD, *n* = 3. (**B**) The effect of pH-sensitive liposomes enriched with CL on the amount of DNR retained by B16-BL6 cancer cell lines. * Indicates *p* < 0.05. Values represent mean ± SD, *n* = 3.

Figure 4A demonstrates the effects of F1 on cellular DNR retention in the tumor cells. The decline in the cellular DNR levels, as a result of drug efflux into fresh EBSS medium, is presented as a function of time up to 4 h. The F1 liposomes demonstrated enhanced DNR cellular retention compared with the DNR solution and F2 (*p* < 0.05). After 2 h, F1 enhanced the DNR retention by 3-fold and 2.2-fold compared with the DNR solution and F2, respectively (Figure 4B). There was no significant difference in the retention between F2 and free DNR (*p* > 0.05).

The limited availability of anthracycline drugs due to their insufficient distribution in solid tumors in association with efflux by the P-gp pump increases sequestration in endosomes and tumor cell packing density [63]. Anthracycline DOX retention was significantly greater when more DOX accumulated inside cancer cells because the P-gp pump is saturated by a high drug concentration [64]. In our study, the higher cellular retention of DNR was due to an increase in passive drug diffusion. Since CL increases the membrane fluidity and affects its mechanical properties, it allows more DNR to accumulate inside tumor cells, which causes the saturation of the P-gp pump and decreases drug efflux. Furthermore, DNR accumulation in a large amount inside tumor cells will limit the drug’s ability to move outside cells across the destabilized membrane. DNR will accumulate strongly in the nucleus and acid vesicles (more intracellular DNR store). Such strong binding will decrease the efflux rate and cause a low diffusion coefficient [65].

**Figure 5:**
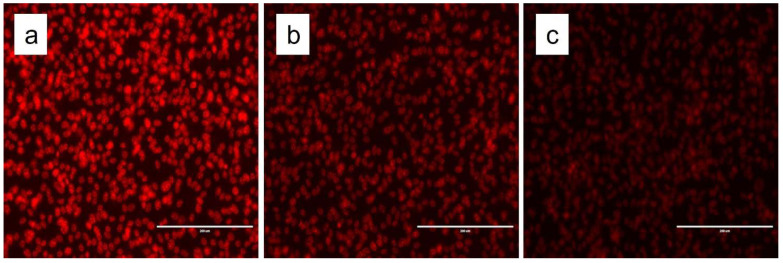
Fluorescence microscopy showing CL enhanced DNR uptake from liposomes F1 (**a**), F2 (**b**), free DNR (**c**).

**Figure 6:**
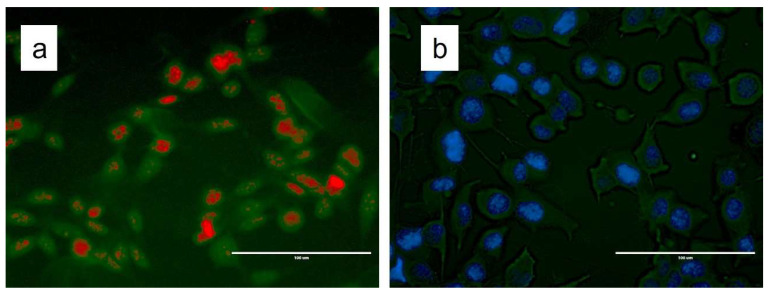
Fluorescence microscopy showing CL interacting with the cellular membrane. Fluorescent CL liposomal formulation encapsulated with DNR (**a**) or no DNR (**b**). Red represents DNR while green represents fluorescent CL. Blue represents nuclei of cells stained with DAPI while green represents fluorescent CL.

We visualized the uptake of DNR and liposomal formulations by fluorescence imaging. Figure 5 shows that F1 displayed significantly higher DNR accumulation. After 6 h of incubation (14 μM DNR), the fluorescence levels were consistently higher in the formulation enriched with CL compared with F2 and free DNR. The results correlate with both cytotoxicity and cellular uptake results. Fluorescence microscopy results demonstrated that CL enhanced the delivery of DNR into tumor cells through changes in the physical properties of the cell membrane such as thickness and permeability. To investigate the interaction of CL with the cellular membrane, we incorporated fluorescent CL into the liposomal formulation encapsulated with DRN. Furthermore, we created a blank liposomal formulation with fluorescent CL. As shown in Figure 6, CL interacted with the membrane bilayer, allowing more DNR to accumulate in the nucleus.

The physical stability of different liposomes during storage (4 °C for one month) was followed by measuring time-dependent changes in liposome size, EE%, DL%, zeta potential, and the polydispersity index (Table 3). There were no significant changes in any parameters during the stability study.

In order to develop stable pH-sensitive liposome formulations, CHEMS was added to the lipid composition to increase the membrane rigidity. It is usually found that between 20 and 50 mol% of cholesterol is required to maintain bilayer stability when mixed with HII-preferring lipids such as DOPE [66]. CHEMS is an acidic cholesterol ester that self-assembles into bilayers in the neutral aqueous media and is commonly employed in mixtures with dioleoylphosphatidylethanolamine (DOPE) to form ‘pH-sensitive’ fusogenic vesicles. In neutral pH, it functions as cholesterol and will cause the liposome to be rigid. Furthermore, it stabilizes DOPE. Besides, the incorporation of PEG-PE in the membrane of pH-sensitive liposomes imparts steric stability to these liposomes [67]. Since anthracycline drugs precipitate as fibrous-bundle aggregates in the liposomes [68], a high drug:lipid ratio might cause liposomal deformation. The drug:lipid ratio of 1:5 used in our formulations did not cause liposomal membrane deformation, which explains the good stability profiles of liposome formulation, especially in terms of EE.

## 4. Conclusions

The optimal liposome formulation had a 40:30:5:17:8 molar ratio for DOPE/cholesterol/DSPE-mPEG (2000)/CL and SA. The liposomes prepared at a drug-to-lipid molar ratio of 1:5 exhibited high drug encapsulation efficiency (>90%), small size (~94 nm), and narrow size distribution (~0.16). DNR was rapidly released from pH-sensitive liposomes under an acidic environment (within 1 h), while under physiological pH, liposomes exhibited a good stability profile. The pH-sensitive liposomes exhibited a higher cytotoxic and DNR cellular uptake effect on B16-BL6 cell lines than liposomes similar to DaunoXome^®^ and free DNR, suggesting that pH-sensitive liposomes change the physical properties of the plasma membrane leading to more diffusion of DNR into cancer cells. Therefore, this formulation appears to be a promising delivery system for the treatment of melanoma.

## Figures and Tables

**Table 1:** Composition and molar ratio of various liposomal formulations.

Ingredients	F1	F2 *	F3 ^#^
DOPE	40	-	40
CHEMS	30		30
DSPE-mPEG (2000)	5	-	5
DSPC	-	10	-
Cholesterol	-	5	-
CL	17	-	17
SA	8	-	8
DNR:lipid ratio	1:5	1:10:5	-

* Liposomal formulation similar to DaunoXome^®^. 1:10:5 is the molar ratio of daunorubicin:DSPC:cholesterol. 1:5 is the molar ratio of daunorubicin:total lipids. ^#^ Blank formulation, used in the cytotoxicity studies.

**Table 2:** Physicochemical characteristics of different liposome formulations. Values are expressed as mean ± SD, *n* = 3.

DNR Liposomal Formulation	Encapsulation Efficiency %	Drug Loading (%)	Particle Size (nm)	Polydispersity Index	Zeta Potential (mV)
F1	95.0 ± 3.7	15.8 ± 0.6	94.0 ± 3.7	0.16 ± 0.03	−39.1 ± 3.1
F2	94.0 ± 0.5	15.8 ± 0.4	83.0 ± 3.1	0.18 ± 0.07	−5.0 ± 1.8

**Table 3:** Stability of formulations stored at 4 °C under N_2_ and protected from light for 1 month. Values represented as mean ± SD, *n* = 3.

	F1		F2	
Formulation	Initial	1 month	Initial	1 month
Size Particle Size (nm)	94.0 ± 3.7	112.3 ± 4.2	83.0 ± 3.1	86.0 ± 3.2
PI	0.16 ± 0.03	0.18 ± 0.01	0.18 ± 0.07	0.23 ± 0.07
EE %	95.0 ± 3.7	86.0 ± 4.4	94.0 ± 0.5	92.0 ± 2.7
DL %	15.8 ± 0.6	14.7 ± 0.8	15.8 ± 0.4	16.6 ± 0.3
Zeta Potential (mV)	−39.1 ± 3.1	−36.4 ± 3.9	−5.0 ± 1.8	−4.0 ± 1.9

PI = Polydispersity Index; EE% = Encapsulation Efficiency; DL% = Drug Loading.

## Data Availability

Not applicable.
